# Translation of biophysical environment in bone into dynamic cell culture under flow for bone tissue engineering

**DOI:** 10.1016/j.csbj.2023.08.008

**Published:** 2023-08-17

**Authors:** Shuntaro Yamada, Philipp Niklas Ockermann, Thomas Schwarz, Kamal Mustafa, Jan Hansmann

**Affiliations:** a Center of Translational Oral Research-Tissue Engineering, Department of Clinical Dentistry, Faculty of Medicine, University of Bergen, Norway; b Fraunhofer Institute for Silicate Research ISC, Translational Center Regenerative Therapies, Germany; c Chair of Tissue Engineering and Regenerative Medicine, University Hospital Würzburg, Germany; d Department of Electrical Engineering, University of Applied Sciences Würzburg-Schweinfurt, Germany

**Keywords:** Bioreactor, Bone regeneration, Tissue engineering, Mesenchymal stem cells, Dynamic cell culture, Osteogenic differentiation

## Abstract

Bone is a dynamic environment where osteocytes, osteoblasts, and mesenchymal stem/progenitor cells perceive mechanical cues and regulate bone metabolism accordingly. In particular, interstitial fluid flow in bone and bone marrow serves as a primary biophysical stimulus, which regulates the growth and fate of the cellular components of bone. The processes of mechano-sensory and -transduction towards bone formation have been well studied mainly in vivo as well as in two-dimensional (2D) dynamic cell culture platforms, which elucidated mechanically induced osteogenesis starting with anabolic responses, such as production of nitrogen oxide and prostaglandins followed by the activation of canonical Wnt signaling, upon mechanosensation. The knowledge has been now translated into regenerative medicine, particularly into the field of bone tissue engineering, where multipotent stem cells are combined with three-dimensional (3D) scaffolding biomaterials to produce transplantable constructs for bone regeneration. In the presence of 3D scaffolds, the importance of suitable dynamic cell culture platforms increases further not only to improve mass transfer inside the scaffolds but to provide appropriate biophysical cues to guide cell fate. In principle, the concept of dynamic cell culture platforms is rooted to bone mechanobiology. Therefore, this review primarily focuses on biophysical environment in bone and its translation into dynamic cell culture platforms commonly used for 2D and 3D cell expansion, including their advancement, challenges, and future perspectives. Additionally, it provides the literature review of recent empirical studies using 2D and 3D flow-based dynamic cell culture systems for bone tissue engineering.

## Graphical Abstract

**Figure:**
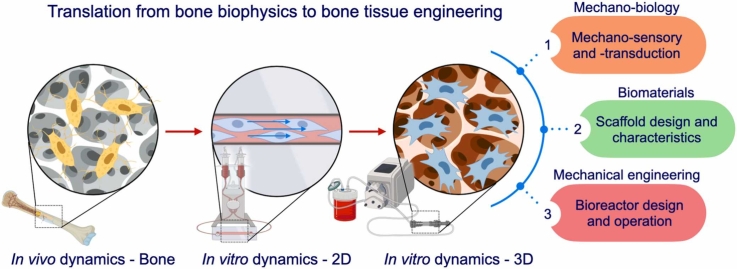

## 1 Introduction

Bone tissue engineering is an emerging field of research that aims to regenerate a functional bone tissue [1]. In the realm of successful tissue regeneration, a widely acknowledged strategy involves the utilization of three-dimensional (3D) tissue-engineering constructs, which encompass a combination of scaffolding biomaterials, multipotent stem cells, and growth factors [2]. The overarching aim is to accurately replicate the geometric attributes and functional aspects of the targeted tissues. Furthermore, the adoption of 3D cell culture methodologies can profoundly enhance cellular functionalities [3]. This is in stark contrast to the limitations posed by conventional 2D monolayered cultures, which have the potential to compromise the innate cellular capacities for growth and differentiation [3]. For effective bone regeneration, 3D scaffolds with pores ranging from 100 to 800 µm are commonly designed to mimic a trabecular bone structure, proving stem cells geometrical cues to promote osteogenic differentiation [4], [5], [6]. However, scaling up the size of cell-loaded constructs faces a major challenge because the transfer of nutrients and gases within 3D scaffolds is often limited to the surfaces due to the constraints of passive diffusion, which leads to the formation of the necrotic core inside the constructs [7]. Therefore, using a 3D dynamic cell culture platform with medium agitation or perfusion is a preferable approach to improve mass transfer while providing biophysical stimuli to the cells.

Developing viable tissue-engineered constructs requires an in-depth understanding of the complex biophysical environment of targeted tissues for regeneration. In bone, cellular components, namely osteocytes, osteoblasts, osteoclasts and their progenitor cells, exhibit mechanosensory features, regulating bone homeostasis and repair by adapting to biophysical stimuli from physical activities or disuse [8]. Similarly, mesenchymal stem cells (MSCs) also possess the characteristics, and it has been proven that appropriate mechanical stimuli may trigger osteogenic differentiation in vitro by using various dynamic cell culture platforms, often referred to as bioreactors [9]. To identify the optimal biophysical stimuli to the cells during cell culture, knowledge in biophysics in the native tissue needs to be translated into the application. This review aims to emphasize the importance of integrating bone biophysics and engineering techniques into bone tissue engineering for the development of effective bone regeneration strategies using 3D dynamic cell culture systems. Additionally, it reviews recent studies that have used these dynamic cell culture systems, particularly utilizing fluid stimuli, to investigate cellular responses to biophysical cues and optimize the tissue engineering strategies for bone regeneration.

## 2 Biophysical environment in bone and bone marrow

The primary function of bone is to provide structural support against loads. However, various factors such as diseases, injury, sedentary lifestyle, microgravity in space, and aging can lead to decreased physical activity, resulting in bone remodeling that favors resorption, whereas moderate time-varying loads promote bone accretion [10]. Bone deformation caused by reaction force from the ground and muscle contraction can be explained as compressive, tensile, tortional and shear strains [11]. *In vivo* measurement by implanting strain gauges demonstrated that light exercises such as walking or jogging generated peak strain of approximately 200 με up to 1000 με in human tibias [12]. Strenuous activities, such as uphill and downhill zigzag running, recorded a 3-fold increase in strain compared to that during walking, but peak strain was maintained below 2000 με regardless of activity type [13]. In 1987, Frost H. M. postulated that balanced bone homeostasis requires functional strain within the range of 300 με to 1500 με, and strain below and above this optimal range may lead to bone resorption [14]. This theory has been well supported to date [15].

**Fig. 1:**
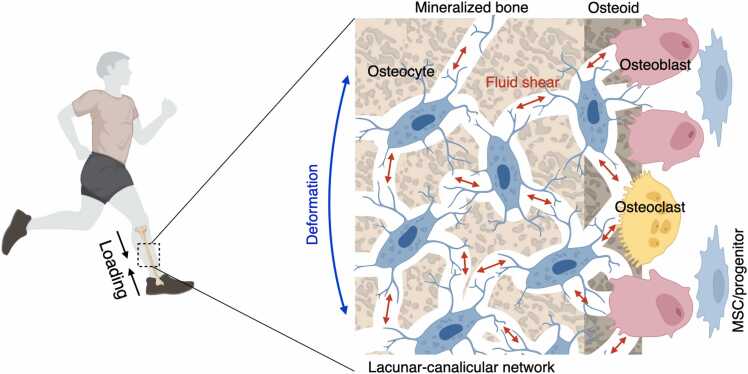
Fluid shear stress generated by physical activity in a lacunar-canalicular network. Osteocytes are primarily responsible for detecting loading force on a bone, which occurs via the detection of alterations in interstitial fluid flow within the lacunar-canalicular network. Subsequently, these mechanical signals are converted into biochemical signals that are then propagated to neighboring cells. Adapted from Qin et al. (2020).

**Fig. 2:**
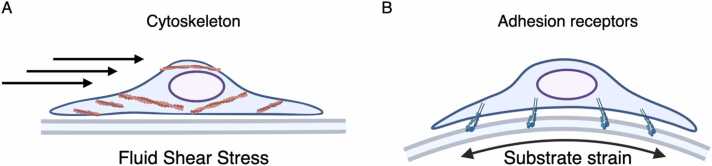
The perception of shear stress and substrate strain stimuli by cells. (A) The surface and sub-membrane region of cells experience the application of shear stress through cytoskeleton, while (B) cell-matrix adhesion receptors primarily perceive substrate strain. Adapted from Mullender et al. (2004).

The major biophysical stimuli in bone at a micro scale are considered to be fluid shear stress and substrate strain, which are likely to act in combination rather than independently [16]. The interstitial fluid, which accounts for approximately 20% of body weight, plays a crucial role in transporting nutrients, gases, and waste products to and from cells through extracellular matrix (ECM) [17]. In comparison to other tissues, interstitial fluid within a lacunar-canalicular network of bone exhibits a higher flow velocity due to the confinement imposed by the surrounding mineralized tissue [8], [18], [19], [20]. Mechanical loading generates pressure gradients that drive the flow of interstitial fluid from the compressive side of bone to the tensile side [21] (Fig. 1). A numerical model estimated that shear stress in a lacunar-canalicular network may reach around 0.8–3 Pa under physiological loading conditions, and similar stress was also predicted on bone-forming sites where osteoblasts reside [19]. In addition to shear stress, substrate strain is a vital biophysical factor that can influence cellular behavior. The application of complex mechanical loads on bone elicits spatially distributed strains, compressive strain on one side of the substrate and tensile strain on the other, thereby inducing cellular compression or stretching depending on the position of the cells [22]. The mechanical stresses that cells experience due to fluid flow and substrate stain are distinct, albeit overlapping in some respects. While fluid flow generates stress on the cell surface and subcellular regions beneath the unbound cell membrane, substrate strain places mechanical stress on the cell's adhesion receptors, affecting its binding side (Fig. 2 A, B) [22]. Consequently, the extent of cellular deformation, particularly in the innermost regions, varies between these two modes of mechanical stimulation, with fluid flow being a more potent inducer of such deformation. An *in silico* study has shown that physiological fluid shear stress at a magnitude of 0.6 Pa induces approximately 7.5 times greater membrane displacement compared to physiological substrate strain at a magnitude of 1000 με [23]. The study also found that substrate strain at a magnitude of 5000 - 8000 με, which is far beyond the physiological limit, was required to produce similar cellular deformation to fluid shear stress of 0.6 Pa. Cellular deformation has been linked to anabolic responses to stimuli. While fluid shear stress at a range of 0.4–0.6 Pa has been found to increase the production of nitric oxide (NO) and prostaglandin (PG) E2 by osteocytes and osteoblasts in vitro, substrate strain did not have a significant effect on these biomolecules, even at magnitudes of up to 5000 με [23], [24], [25], [26], [27]. Although some studies have suggested that substrate strain within the physiological range can promote osteogenic differentiation, Owan et al. have proven that anabolic response is dependent on the increase in flow velocity caused by substrate displacement (i.e., deformation), rather than the magnitude or rate of the substrate strain itself [28]. Subsequently, tensile and compressive strains with supra-physiological magnitudes of 10,000 - 100,000 με have been frequently used to test osteogenic responses in osteoblast and MSC culture, which is close to or exceeds the threshold for bone fracture strain [29], [30], [31], [32], [33].

In bone marrow, fluid flow is the primary driver of mechanical stimuli, owing to its rich vascular network comprising central venous sinus, nutrient artery, and marrow sinusoids [20]. The magnitude of shear stress in bone marrow is related to its viscosity (i.e., yellow bone marrow or red bone marrow) and trabecular porosity (i.e., healthy bone or osteoporotic bone) [34]. Computational models predicted that high-frequency low-magnitude mechanical stimulation (HFLMMS: 1 g, 30 – 50 Hz) at an amplitude of 1 g/10 µm would inflict shear stress of approximately 0.1–5 Pa in the bone marrow of long bones and vertebrae [35], [36]. An *ex vivo* study using porcine femurs found that compression at a magnitude of 0.07–5 kPa, similar to physiological loading in humans, generated shear stress at approximately 1.5–25 Pa in the bone marrow [37].

The interdependence of fluid shear stress and other biophysical stimuli complicates their distinction. Nonetheless, based on the available evidence, it is reasonable to conclude that fluid shear stress serves as a primary biophysical stimulus both in bone and bone marrow that initiates bone remodeling reactions by stimulating the residing cells.

## 3 Mechanosensation and mechanotransduction in bone metabolism processes

The process of bone remodeling involves the coordinated adaptation of osteocytes, osteoblasts, osteoclasts, and their progenitors in response to mechanical stimuli. Communication between these cells occurs through the lacunar-canalicular network, in which osteocytes are connected via dendritic processes that form a syncytium toward bone surfaces and bone marrow where osteoblasts, osteoclasts, and their progenitors reside [38]. Upon mechanosensation, the biophysical signals are converted to biochemical signals, a process called mechanotransduction, which are then transmitted mainly through gap junctions [39].

Osteocytes, which make up over 90% of total bone cells and are approximately ten times more populous than osteoblasts, serve as the central command for mechanosensation and mechanotransduction processes in bone [40], [41]. They perceive biophysical signals through various mechanisms, including their cytoskeletons, dendritic processes, primary cilia, focal adhesions, ion channels, and surface mechanoreceptors such as integrins and cadherins [42]. In response to mechanical stimuli, osteocytes release anabolic signals such as NO and PG, which activate the canonical Wnt signaling pathway in particular [43], [44]. The activation of the signaling induces the expression of its target genes including a key transcriptional factor for osteogenesis, Runt-related transcription factor 2 (Runx2), to initiate adaptive changes in bone [45], [46]. The importance of osteocytes in bone adaptation to mechanical stimuli was demonstrated by experiments in transgenic mice, in which osteocytes were conditionally ablated using dentin matrix protein 1 (Dmp1) as a specific osteocyte marker [47]. Short-term osteocyte ablation did not affect the population and functionality of osteoblasts or osteoclasts, nor did it alter bone mass. However, osteocyte ablation prevented disuse-induced bone atrophy, while leading to significant bone loss with increased osteoclast number and activity in wild-type mice. On the other hand, load-induced bone formation was not inhibited by osteocyte deficiency, but by osteoblast deficiency [47], [48]. These findings suggest that osteocytes are necessary for disuse-induced bone atrophy by regulating osteoclast activity, while load-driven bone formation can bypass the osteocytes' command.

The mechanosensitive features of osteoblasts are similar to those of their ancestral osteocytes, as they immediately produce NO and PG upon mechanical stimulation in vitro and activate the canonical Wnt signaling pathway, much like osteocytes [45], [49], [51], [50]. However, osteoblasts' mechanosensory role in bone appears to be limited compared to that of osteocytes due to their lower frequency and biased distribution on bone surfaces [27]. Osteoblasts reside on soft osteoid and newly mineralized bone surfaces, where the fluid shear stress exerted on them is estimated to be lower than that experienced by osteocytes in a lacunar-canalicular network [52]. A computational model of load-induced trabecular bone remodeling showed that incorporating osteoblast-based surface remodeling had no additional effect on overall bone mass and architecture [53]. Moreover, osteoblasts' sensitivity to mechanical stimuli is known to be inferior to that of osteocytes, leading researchers to consider them an auxiliary entity in mechanosensation in bone [27].

Bone marrow-derived MSCs (BMSCs), along with other bone-forming cells, play a supportive role in mechanically induced bone remodeling despite being relatively rare [54]. Nestin-positive perivascular stromal cells have been identified as BMSCs in the bone marrow niche, capable of differentiating into multiple mesenchymal lineages when grown in an adherent culture [55]. These cells alter their phenotype in response to the mechanical environment. The response of BMSCs to mechanical stimuli in vivo has been demonstrated in various rodent models. For example, moderate training on a treadmill or engaging in climbing exercises has been shown to enhance the colony-forming ability of BMSCs, with the upregulation of anti-apoptosis regulators such as Survivin and B-cell lymphoma-2 [56], [57], [58], [59]. In terms of differentiation, it was found that BMSCs in individuals undergoing long-term training upregulated osteogenic markers, such as Runx2, alkaline phosphatase (ALP), and Osteocalcin (Ocn), while downregulating adipogenic markers, such as Peroxisome proliferator-activated receptor gamma (PPARγ), CCAAT/enhancer binding proteins, and fatty acid binding protein [56], [60], [61]. Similarly, HFLMMS stimulated BMSC self-renewal and osteogenic differentiation. HFLMMS applied at 0.2 g, 90 Hz for 15 min per day, 5 days per week, for 6 weeks, increased the number of Stem cell antigen-1 (Sca1/Ly6)-positive stromal cells in bone marrow by approximately 40%, and upregulated Runx2 expression significantly while downregulating PPARγ expression of the stimulated putative BMSCs [62]. The mechanical regulation of BMSCs in vivo involves multiple signaling pathways, with the BMP-Smad and canonical Wnt signaling pathways being crucial mediators of the response of BMSCs to mechanical stimuli [61]. In trained subjects, BMSCs exhibited a significant increase in the phosphorylation of Smad1, a key signal transducer for BMP receptors [61]. However, when a selective inhibitor of the BMP-Smad pathway was administered during exercise, the promotive effects of exercise on BMSC growth and osteogenic differentiation were disproven [61]. Research also suggests that the canonical Wnt signaling pathway participates in in vivo fate determination and favor for osteogenesis [44], [60]. Nevertheless, the regulation of BMSCs by mechanical stimuli in vivo is not fully understood, with the question remaining as to whether mechanical stimuli directly regulate them or indirectly via osteocytes.

## 4 2D dynamic cell culture under flow- studying osteogenic responses of mesenchymal stem cells

**Fig. 3:**
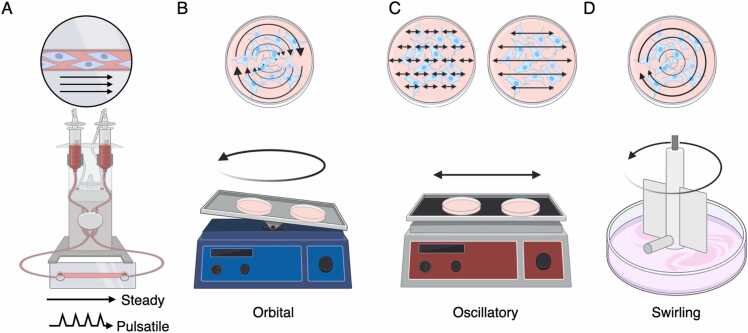
Schematic illustration of the frequently employed 2D dynamic cell culture systems that utilize fluid flow stimuli to study cellular responses. The type of fluid stimuli may be distinguished as (A) steady, pulsatile, (B) orbital, (C) oscillatory, and (D) swirling, depending on apparatus used. Arrow indicates the predicted direction of fluid flow.

Conventional 2D cell culture methods cause a genetic instability, leading to cellular senescence and the reduced differentiation capability of MSCs [63]. This has been considered due to a lack of biophysical as well as geometrical stimuli, which are present in body. The interrelationship between various stimuli is complex and not easy to differentiate in vivo as well as in vitro. Conventional studies have elucidated, however, that fluid shear stress is a primary biophysical stimulus in bone and bone marrow that promotes osteogenesis [20], [28]. As the potential of stem cell therapy grows, researchers have shifted their focus towards controlling the growth and fate of MSCs in culture by applying mechanical stimuli relevant to in vivo since the 2000 s [64]. In this regard, naturally, fluid stimuli have received significant attention, and 2D dynamic cell culture systems employing various flow patterns, such as steady or pulsatile (e.g., by peristaltic pumps or syringe pumps), orbital (e.g., by orbital shakers), oscillatory (e.g., by linear shakers), and swirling (e.g., by spinner flasks or spinning motor) flows, have been frequently utilized (Fig. 3 A-D).

Since the mechanical sensitivity of MSCs was not well understood, compared with osteocytes and osteoblasts, a broad range of fluid shear stress varying from sub-millipascal to supraphysiological levels was tested (Table 1). Briefly, fluid shear stress at physiological magnitudes has a supportive effect on the osteogenic potential of MSCs in vitro. Similar to osteocytes and osteoblasts, the mechanotransduction process in MSCs starts with the rapid production of NO and PGE2 and transient modulation of canonical Wnt signaling in response to fluid shear stress [65], [66], [67]. Subsequently, the cells exhibit osteogenic characteristics, including the upregulation of osteogenic markers and ALP activity, particularly when cultured in an osteoinductive medium (e.g., in the presence of dexamethasone, beta-glycerophosphate, and ascorbic acid). *In vitro* studies showed that BMSCs exposed to fluid shear stress as low as 0.1 mPa under steady flow conditions upregulated the expression of Runx2 mRNA and displayed enhanced ALP activity after 4 days of stimulation [68]. Similar observations were reported at both physiological (i.e., 0.5–2 Pa) and supraphysiological (i.e., up to 10 Pa) magnitudes in steady continuous flow models [68], [69], [70], [71], [72]. However, studies using continuous flow models primarily focused on short-term effects on osteogenic responses (i.e., within an hour to a few days) unless the fluid stimulus was extremely low (i.e., 10 mPa and less) probably due to the inhibitory effect of the stimulus on cell growth. It has been shown that a shear stress of 1 Pa inhibits cell growth while inducing osteogenic responses [68]. This observation is consistent with a study analyzing cell kinetics under flow, which showed that fluid shear stress arrested MSC cell cycle at G0/G1 phases [73]. Given that osteogenic differentiation is a sequential process of cellular events, longer observation periods, possibly more than a week, are particularly valuable. To balance the promotion of osteogenic responses and cell growth, fluid stimuli need to be applied placidly and/or intermittently for long term dynamic cell culture. Intermittent steady flow seems to be more supportive for cell growth and exerts a robust promoting effect on osteogenic differentiation, both in the short-term and long-term, despite shorter stimulation periods per day compared with continuous flow models [68], [74], [75], [76]. Alternatively, more physiological-like conditions can be reproduced by oscillatory or pulsatile models. For example, Lim et al. demonstrated that BMSCs exposed to an oscillatory flow model at a magnitude of 0.5 Hz, 1 mPa, for only 10 min per day, resulted in a significant increase in the expression of osteogenic genes and functionality markers [77]. These findings suggest that even a short-term, subtle fluidic stimulus, which may be significantly weaker than a physiological level, may be sufficient to guide MSCs to the osteogenic lineage in vitro.

**Table 1:** Summary of studies (2010–2022) on the effect of shear stress on osteogenic differentiation compared to the static culture.

Species	MSC source	Substrate	Medium	Flow type	Flow condition	Shear stress	Time point	Growth	Upregulated osteogenic *genes*/PROTEIN	Downregulated osteogenic *genes*/PROTEIN	ALP activity	Calcium deposition	Other	Ref
Rat	Bone marrow	Glass	OM	Steady	2 hr/dayf low between Day 1 and Day 4	0.4 Pa	Day 4 (PCR) Day 21	NA	COL1, OPN	*runx2, bmp2*	NA	- (Day 21)	Laminn A ↑ Factin ↑	[78]
					2 hr/day flow between Day 7 and Day 9	0.4 Pa	Day 10 (PCR) Day 21	NA	*col1,* RUNX2, COL1, OCN, OPN	-	↑	↑ (Day 21)	Laminn A ↑ Factin ↑	
					2 hr/day flow between Day 14 and Day 17	0.4 Pa	Day 17 (PCR) Day 21	NA	*alp, col1, ocn,* RUNX2, COL1, OPN	-	NA	↓ (Day 21)	Laminn A ↑ Factin ↑	
Human	Bone marrow	Well plate	OM	Orbital	6 hr/day	0.3–0.7 Pa	Day 5	NC	NA	NA	↑	NA	Notch signalling ↑	[79]
Mouse	Not specified	Micropatterned well plate	GM	Swirling	1 hr flow-5 hr interval	0.5–0.8 Pa	Day 3	NA	*alp, ocn, col1,* ALP, OCN, COL1	-	↑	NA	YAP ↑ Apoptosis ∝ ALP	[80]
Rat	Bone marrow	Well plate	OM	Oscillatory	1 Hz, 2 hr/day	0.0375 Pa	Day 7	NC	*alp, ocn, runx2*	-	↑	NA	INTB1 ↑ FAK-ERK1/2 pathway↑	[81]
							Day 14	NC	NA	NA	NA	↑		
Human	Bone marrow	Polymer coverslip	GM	Steady	Continuous	10 Pa	Day 1	NA	*dmp1, bmp2, bsp, opn*	-	NA	NA	Hyaluronan synthases ↑	[69]
Rat	Bone marrow	Col-coated glass	OM	Steady	1 h flow-7 hr interval	1 Pa	Day 4	NC	*alp, runx2*	-	↑	NA		[68]
						0.0001 Pa	Day 4	↑	*alp, runx2*	-	↑	NA		
					Continuous	1 Pa	Day 4	↓	NA	NA	NA	NA		
						0.0001 Pa	Day 4	NC	*runx2*	-	↑	NA		
Equine	Adipose	Well plate	GM	Oscillatory	0.67 Hz, Continuous	0.077 Pa	Day 10	↑	-	-	NC	NC		[82]
							Day 21	↑	-	-	NC	NC		
			OM	Oscillatory	0.67 Hz, Continuous	0.077 Pa	Day 10	↑	*alp*	*col1*	↑	NC		
							Day 21	↑	*ocn*	*alp*	↑	↑ (?)		
Rat	Bone marrow	Col-coated glass	OM	Steady	3 day OM induction-20 min flow	1 Pa	Day 3	NA	NA	NA	↑	NA		[83]
Mouse	C3H10T1/2	Fibronectin-coated glass	OM	Oscillatory	2 Hz, Continuous	1 Pa	Hour 2	NA	*cox2, runx2, opn*	-	NA	NA		[84]
					2 Hz, 4 hr/day, Day 1,2,4,5 only	1 Pa	Day 14	NA	COL1	-	NA	NA	Ca2 ^+^ ↑	
Human	Bone marrow	Fibronectin-coated 1 µm wells	OM	Steady	Continuous	1 Pa	Day 2	NA	RUNX2, OPN, ALP, OCN	-	NC	NA	RhoA ↑ Factin↑ CD105↓	[70]
Rat	Bone marrow	Col-coated glass	GM	Steady	Continuous	1 Pa	Hour 1	NA	NA	NA	↑	NA	Factin ↑ Ca2 ^+^ ↑	[71]
Mouse	Bone marrow	Col-coated glass	OM	Steady	40 min flow-10 min interval	1.2 Pa	Hour 3	NA	*sp7, alp, dlx5*	-	NA	NA	RUNX2 -TRPM7-Sp7 pathway ↑	[85]
Human	Alveolar Bone	Well plate	OM	Oscillatory	0.5 Hz, 10 min/day	0.001–0.002 Pa	Day 14	↑ (Day 4)	*runx2, col1, alp, ocn, opn*	-	↑	↑		[77]
					0.5 Hz, 2 hr/day	0.001–0.002 Pa	Day 14	NC (Day 4)	*runx2, col1, alp, ocn, opn*	-	NC	NC		
Human	Bone marrow	Polycarbonate/Glass	GM	Steady	Continuous	2.2 Pa	Day 7	NA	*bmp2, bsp*	-	NC	NA		[72]
			OM	Steady	Continuous	2.2 Pa	Day 7	NA	*opn*	-	NC	NA		
Human	Dental Pulp	Polylysine-coated glass	GM	Pulsatile	5 Hz, Continuous	0.6 Pa	Hour 1	NA	NA	NA	NA	NA	NO ↑ PGE2 ↑	[66]

Search: ((Fluid shear stress) AND (Mesenchymal stem cells [MeSH Terms]) AND (Osteogenic OR Osteogenesis [MeSH Terms] OR (lineage specification))

GM: Growth medium, OM: Osteogenic medium, NA: Not assessed, NC: No change

## 5 3D dynamic cell culture under flow - tissue engineering approach for bone regeneration

The studies using the 2D systems have proven that MSC growth and fate may be controlled mechanically in vitro. The knowledge has been translated into the field of tissue engineering in regenerative medicine, whose object is to produce functional tissue-like constructs that can regenerate damaged tissues in the body. For bone regeneration, 3D scaffolds, on/in which MSCs are seeded, are designed to imitate the trabecular-like porous architecture of bone tissue. Scaffolds provide a biomimetic environment where cell-cell and cell-matrix interactions occur in a more physiological manner compared to monolayered cell culture [86]. However, 3D cell culture poses a challenge to ensure homogeneity inside the engineered constructs due to the low permeability of the scaffolds, which may result in a non-uniform distribution of cells, leading to competition for nutrients during culture [7], [87] The interior of scaffolds may become an unsuitable environment for cells due to a lack of nutrients and oxygen, hindering their scaling up in size for clinical application [88]. To overcome this challenge, the development of appropriate 3D dynamic cell culture platforms, often referred to as bioreactors, has been in high demand as a competitive alternative to conventional static culture.

Tissue engineering bioreactors are devices that provide supportive culture conditions for cells in/on 3D scaffolds by aiding their biological processes [89]. Bioreactors are designed based on several key development concepts that often involve the convergence of science and technology across multiple disciplines although bioreactors tend to be regarded as “black boxes” developed through trial and error [90]. At a minimum, bioreactors must provide a suitable environment for cell growth, including maintaining sterility, controlling temperature and humidity, providing appropriate aeration, and supplying necessary nutrients to the cells [91], [92]. Additionally, to address the diffusive limitation of nutrients, gasses, and waste products within the constructs, bioreactors designed for bone tissue engineering often incorporate mechanisms to generate medium movement, such as perfusion or agitation [93], [94]. For example, when a porous scaffold with an average pore size of 200 µm is used, the diffusion limit is estimated to be 200–300 µm from the surface under static conditions [7]. If the distance exceeds this limit, cell necrosis may occur in the innermost part of the scaffolds due to inadequate supply of nutrients and gasses. Thus, this emphasizes the importance of dynamic strategies to enhance mass transfer into 3D constructs instead of relying solely on passive diffusion driven by concentration gradient. To enhance mass transfer, a variety of flow bioreactors have been developed for successful tissue engineering applications. Spinner flask bioreactors, rotating wall vessel bioreactors, and perfusion (laminar flow) bioreactors are the most commonly used flow bioreactors [91].

**Fig. 4:**
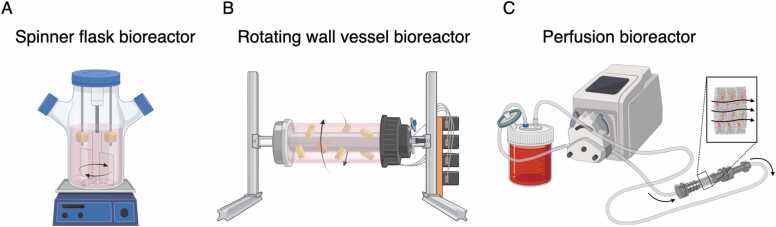
Schematic illustration of flow-based bioreactor systems commonly used for tissue engineering for bone regeneration. (A) A spinner flask and (B) a rotating wall vessel bioreactor are used for medium agitation, while (C) a perfusion bioreactor generates a laminar flow passing through the scaffolds placed in the system.

Spinner flask bioreactors are simple agitation systems with a glass flask, a filter cap to exchange gas, and an integrated stirrer or a magnetic stirrer placed at the bottom of the flask to generate swirling flow (Fig. 4A). In the flask, cell-loaded constructs are immobilized in needles or sample holders. As a consequence of swirling the cell culture medium, homogenous concentration in the cell culture medium and at the interface to the tissue are maintained. Thereby, accumulations of waste products and low concentrations of nutrients and oxygen at the liquid-scaffold interface are eliminated. This concept supports mass transport across the scaffold boundaries. It was reported that the proliferation and distribution of MSCs in hydroxyapatite (HA) scaffolds were improved in a spinner flask bioreactor [95]. Notably, a supportive effect on cell proliferation was more remarkable in scaffolds with larger (i.e., 500 µm) pores than those with smaller (i.e., 200 µm) pores, but stimulatory effect on osteogenic differentiation was more distinguished in scaffolds with smaller (i.e., 200 µm) pores. This suggests that mass transfer in the scaffolds is more likely to be improved with larger pore sizes and interconnected porous characteristics, but fluid shear stress exerted on the surfaces can be greater with smaller pore sizes, giving mechanical stimuli to provoke their osteogenic responses. However, a significant drawback of spinner flask bioreactors is that the current generated by the stirrer creates large, nonhomogeneous fluid shear stress that varies spatiotemporally, and produces a transient turbulent flow that may damage cells [96], [97].

Rotating wall vessel bioreactors were initially developed by NASA to study cell and tissue responses in microgravity environments (Fig. 4B) [98]. Compared to spinner flask bioreactors, rotating wall vessel bioreactors generate uniform and homogeneous flows [97]. In this system, cell-loaded samples are placed in a cylindrical chamber that rotates along the long axis. In bone tissue engineering, it has mostly been used for cell cultivation with microcapsules, microbeads, or microspheres. For example, Qiu et al. used the system as an effective means of loading cells onto microspheres, showing that BMSC co-cultured with ceramic microspheres in the bioreactor were able to attach and form ECM on the microspheres homogeneously [99]. The advantage of a rotating wall vessel bioreactor in osteogenesis was shown by Yu et al., demonstrating the significant improvement of matrix mineralization, ALP activity, and Ocn and Osteopontin (Opn/Spp1) expression in osteoblasts grown on poly(D,L-lactic-co-glycolic acid) (PLGA) microspheres [100]. In rotating wall vessel bioreactors, samples are subjected to both microgravity and fluid flow [101]. A previous study reported the suppression of BMSC osteogenic functionalities due to reduced metabolism and the downregulation of the mechanotransduction pathway in an empirical microgravity environment [102], [103]. This provides the insight that the improvement of cellular functionalities observed in rotating wall vessel bioreactors is mainly attributed to fluidic stimulation and improved nutrient distribution by removing static layer on the material surfaces, rather than microgravity. Nevertheless, the reported finding suggests that, in contrast to spinner flask bioreactors, the presence of nutrient concentration gradients at the interior of the scaffolds was not fully resolved [104]. When it comes to the application of macroscale scaffolds in the systems, the limitation of the system for bone tissue engineering becomes noticeable. In a comparative study, the cell proliferation, ALP activity, and calcium deposition of BMSC on porous PLGA scaffolds with a dimension of 12.7 mm × 6 mm were found to be significantly lower in a rotating wall vessel bioreactor, but higher in a spinner flask bioreactor, compared to static culture [104]. The adverse impact is likely to be attributable to the collision of the scaffolds with the bioreactor walls, resulting in the physical damages of the cells [91]. Consequently, large macroscale scaffolds with high mass may not be appropriate for use in rotating wall vessel bioreactors.

Perfusion bioreactors are gaining the greatest popularity in bone tissue engineering due to their potential advantages over other aforesaid concepts (Fig. 4C). Unlike the other systems, perfusion bioreactors can generate laminar flows that pass through the constructs directly, resembling the flow of blood and interstitial fluid. This unique feature provides a significant advantage in nutrient and gas transport, particularly for long-term cell culture with low-diffusive scaffolds [104]. In perfusion bioreactors, flow characteristics such as magnitude, duration (e.g., continuous or intermittent), direction (e.g., steady or oscillatory), and frequency (e.g., pulsatile flow) can be precisely adjusted, leading to predictable bioreactor operation [91]. Studies have demonstrated that perfusion culture can modulate the osteogenic properties of various stem cells through fluid shear stress and improve cell seeding efficiency and distribution within porous scaffolds fabricated in diverse techniques [105], [106], [107], [108], [109], [110], [111], [112], [113], [114], [115], [116], [117], [118], [119], [120], [121], [122], [123], [124], [125], [126], [127], [128], [129], [130], [131], [132], [133], [134], [135], [136], [137] (Table 2). Notably, a study showed that BMSCs on 3D porous scaffolds under perfusion culture localized Runx2 in the nuclei with enhanced ALP activity and ECM formation, proving that the osteogenic differentiation of BMSCs may be induced solely by using a perfusion system without the use of osteoinductive supplements (i.e., dexamethasone, glycerophosphate, and ascorbic acid) [106]. Interestingly, a recent study showed that osteogenic profile induced mechanically by fluid flow noticeably differed from that induced pharmacologically [138]. Under flow, BMSCs exhibited a distinctive profile with concurrent upregulation of ECM proteins and enzymes responsible for ECM degradation, including metalloproteases, indicating the presence of dynamic ECM remodeling processes. This suggests that the use of the bioreactor may offer osteogenic stimuli to the cells in a more “biomimetic” manner compared with conventional pharmacological induction. Noteworthily, the degree of flow-induced osteogenic responses tends to exhibit an inverse relationship with cell proliferation in 3D systems, similar to their 2D counterparts, necessitating careful consideration of the balance between differentiation and growth from a clinical translational perspective [68], [106], [127]. Another advantageous aspect of employing perfusion bioreactors is their potential to serve as an alternative to animal models. The utilization of perfusion bioreactors has the potential to decrease the necessity for animal experimentation. Their heightened controllability and predictability enable a more accurate replication of biophysical stimuli, closely resembling those observed in vivo [139]. In biomaterial research, for example, the degradation profile of biomaterials under a perfusion-based dynamic environment was reportedly more compatible with in vivo observations than under static experimental conditions [140]. Hence, the use of perfusion systems can allow for the assessment of long-term cell-matrix interactions in a physiologically relevant context while minimizing the need for animal testing.

**Table 2:** Summary of studies (2010–2022) on 3D dynamic cell culture using perfusion bioreactors for bone tissue engineering compared to the static culture.

Species	MSC source	Material	Scaffold	Medium	Flow type	Flow condition	Shear stress range	Time point	Growth	Upregulated osteogenic *genes*/PROTEIN	Downregulated osteogenic *genes*/PROTEIN	ALP activity	Calcium deposition	Other	Ref
Human	Bone marrow	Geratin-Alginate	3D printed	OM	Steady	Contiuous, 7 ml/min	10–100 mPa	Day 21	NA	NA	NA	NA	↑		[128]
Human	Adipose	Bio-Oss®	Blocks	GM	Oscillatory	0.00167 Hz, 0.5 ml/min	NA	Hour 1	NA	NA	NA	NA	NA	Cell distribution↑	[132]
Rat	Bone marrow	LTMC	Salt-leached	GM	Steady	8 hr/day, 1.6 ml/min	< 13.35 mPa (mean: 0.40 mPa)	Day 21	↓	*runx2, sp7, bsp, alp, spp1, ocn,* RUNX2, COL1	-	NC	↑		[92]
Human	Bone marrow	Col	Sponge	CM	Steady	3 ml/min overnight followed by 0.3 ml/min	NA	Week 5	NA	NA	NA	NA	↑	Cell distribution↑ Thicker mineralised layer	[117]
Human	Adipose	gelatin-βTCP	Form-casting	OM	Steady	Continuous, 1 ml/min	NA	Day 16	NA	*runx2, ocn,* COL1, ALP	-	↑	NA		[133]
Human	Bone marrow	Chitosan-graphene	Freeze-dried	OM	Steady	1 hr/day, 1 ml/min + 1% axial deformation (1 Hz)	Mean: 0.001 mPa	Day 7	↑	NA	NA	NA	↑		[134]
Human	PT-2501	PLA-nHA	Freeze-dried	GM	Steady	1.21 ml/min for 20 hr followed by 0.3 ml/min	NA	Day 21	↑	*runx2, sp7, alp, col1, spp1, ocn,*	-	NA	↑		[135]
Rabbit	Bone marrow	Col-HA	Freeze-dried	GM/OM	Steady	4 hr/day, 10 ml/min + Mmagnetic field 15 Hz, 1 mT	NA	Day 14	NA	*runx2, col1, ocn*	-	↑	NA	in vivo bone formation↑ *wnt1, lrp6, βcatenin*↑	[136]
Porcine	Bone marrow	Col-PLA-CaP	Freeze-dried	GM	Steady	Continuous, 0.03 ml/min for 6 hr followed by static culture 1–2 ml/min	NA	Day 7	↑	NA	NA	NA	NA	Cell distribution↑	[137]
Human	Adipose	PCL-nHA	Electrospun	OM	Steady	Continuous, 2, 4,5 ml/min	NA	Day 14	↓	*runx2*	*-*	↑	↑		[141]
Rat	Bone marrow	Fibrin	Beads	OM	Steadt	Continuous, 10 ml/min	NA	Day 14	NA	NA	NA	NA	↑	in vivo bone formation↑	[107]
Human	Umbilical cord	PLA-PEG	Salt-leached	OM	Steady	Continuous, 3.47 ml/min	NA	Day 21	↑	RUNX2, COL1, OCN	-	↑	↑		[108]
Human	hES	Gelatin-coated PU	Not specified	OM	Steady	Continuous, 3.47 ml/min	NA	Day 10	↑	NA	NA	↑	NA		[109]
Human	Bone marrow	PLG-HA	Salt-leached	OM	Steady	Continuous, 3 ml/min	NA	Day 21	↓	OCN	*ibsp*	NA	↑	*Compared to culture on orbital shaker	[110]
Human	Bone marrow	PLCL	Salt-leached	OM	Steady	Continuous, 1.6 ml/min + 1–2% axial deformation (1–2 Hz)	0.125–0.175 mPa	Day 2	NA	*spp1, sparc, col1, alp, bmp2*	-	NA	NA		[111]
Rat	Bone marrow	PLCL	Salt-leached	Not specified	Oscillatory	0.5 Hz, 10 ml/min + 60 mmHg hydraulic pressure (0.5 Hz)	NA	Day 14	↑	ALP, OCN	-	NC	↑		[112]
Human	Bone marrow	Silk fibroin	Salt-leached	OM	Steady	Continuous, 0.2 ml/min	< 0.39 mPa	Day 40	↑	NA	NA	↓	↓		[113]
						Continuous, 12 ml/min	< 24 mPa	Day 40	↑	NA	NA	↑	↓		
Human	Adipose	Decellularized bone	Granules	OM/GM	Steady	Continuous, 0.6 ml/min	< 4 mPa	Day 21	NC	NA	NA	↑	NA	Viability decreased in flow rate-dependent manner	[114]
Human	Bone marrow	Col-Alginate	Casted	OM	Steady	Continuous, 3 ml/min	NA	Day 7/14	NA	*bmp2,* BMP2	-	NA	NA	*Co-culture with HUVEC	[115]
Rat	Bone marrow	Col-HA coated decellularized bone	Block	GM	Oscillatory	0.0167 Hz, 1 ml/min 30 min/day	NA	Day 21	NA	OPN, OCN	-	NA	↑ (Day 7)		[102]
Human	hMPC 32 F	Gelatin-coated PU	Foam	OM	Steady	2.5 ml/min, 2 hr on Day 3, 5, and 7	NA	Day 8	↓	NA	-	↓	NA	PGE2↑ *mRNA measured at 1 hr post-perfusion	[118]
						2.5 ml/min, 2 hr on Day 5 only	NA	Day 8	↓	*bmp2, runx2*	-	↓	NA		
						2.5 ml/min, 2 hr on Day 7 only	NA	Day 8	NC	*bmp2, runx2*	-	NC	NA		
						2.5 ml/min, 2 hr on Day 15 only	NA	Day 21	NA	*bmp2, runx2, ocn*	-	NA	↑		
Human	Bone marrow	PLCL	Salt-leached	OM/GM	Steady	1.6 ml/min for 1 hr followed by 0/8 ml/min for 4 × 5 min/hr	Mean: 0.076 mPa	Day 7	NA	*alp, col1, runx2, spp1* (Day 1), *col1, spp1* (Day 7)	-	NA	↑	Cell distribution↑	[119]
Sheep	Bone marrow	CaP-coated TiAl6V4	Mesh	OM	Steady	Continuous, 0.75 ml/min	NA	Day 14	↑	NA	NA	↑ (Day7, 14)	NA		[120]
		HA	Granules	OM	Steady	Continuous, 0.75 ml/min	NA	Day 14	↑	NA	NA	↑ (Day4, 7)	NA		
Rat	Bone marrow	Col-glycoaminoglycan	Freeze-dried	OM	Steady	1 ml/min for 1 hr followed by 0.05 ml/min for 7 hr	< 0.09 Pa	Day 2	NA	NA	NA	NA	NA	*pgf*, *nox1*↑	[121]
Goat	Bone marrow	Starch-PCL	Melt-spun	OM	Steady	Continuous, 1 ml/min	NA	Day 14/21	↓	NA	NA	↑	NA	Viability↓ ECM formation↑	[122]
Human	Bone marrow	Chitosan-aldinate	Beads	CM	Steady	Continuous, 1 ml/min	NA	Day 28	NA	NA	NA	NA	NA	COL2↑ Chondrogenesis↑	[123]
Human	Adipose	Sponceram®	Block	GM	Steady	Continuous, 1 ml/min	Mean: 0.467 mPa	Day 60	NC	*spp1, ocn*	-	NA	↑?		[124]
Rat	Bone marrow	Polyamide-nHA	Sponge	OM	Steady	Continuous, 2 ml/min	NA	Day 21	↑	OCN	-	↑	NA		[125]
Human	hES (H9)	Decellularized bone	Block	OM	Steady	Continuous, 3.6 ml/min	Mean: 6 mPa	Week 5	↑	OPN	-	↑	↑	in vivo bone formation↑	[126]
Rat	Bone marrow	PLG	Salt-leached	OM	Steady	Continuous, 3.9 ml/min	26.4 mPa	Day 15	NC	*opn*, OPN	*ocn*	↑?	NA	PGE2↑	[127]
					Pulsatile	0.017 Hz, 3.1–6.1 ml/min (Mean: 3.9 ml/min)	21–42 mPa	Day 15	↓?	*opn*, OPN	*ocn*	↑?	NA	PGE2↑	
					Pulsatile	0.083 Hz, 3.1–6.1 ml/min (Mean: 3.9 ml/min)	21–42 mPa	Day 15	↓?	*opn*, OPN	*ocn*	↑?	NA	PGE2↑	
Human	Bone marrow	Pro Osteon® 200 µm pores	Block	OM (VitD_3_)	Steady	Continuous, 0.1 ml/min	NA	Day 21	↓	*alp, spp1* (Day 14), *opn* (Day 21)	*runx2, col1, ocn, bsp* (Day 14, 21)	↑(Day 7) ↓(Day 14, 21)	NA	No cell growth in dynamic culture with < 200 µm pores Rounded morphology↑	[129]
		Pro Osteon® 500 µm pores	Block	OM (VitD_3_)	Steady	Continuous, 0.1 ml/min	NA	Day 21	↓	*spp1* (Day 14), *opn, bmp2* (Day 21)	*alp, runx2, col1, ocn, bsp* (Day 14, 21)	↓(Day7–21)	NA		
Rat	Bone marrow	RGD modified PLA	Salt-leached	OM	Steady	Continuous, 0.1 and 1 ml/min	NA	Day 8/16	↑(Day8) ↓(Day 16)	NA	NA	↑	↑		[130]
Human	Bone marrow	Agalose	Casted	CM	Steady	Continuous, 400 µm/s	NA	Week 5	NC	NA	NA	NA	NA	GAG↑ Col2↑ Cell distribution ↑	[131]
		Decellularized bone	Block	OM	Steady	Continuous, 400 µm/s	NA	Week 5	NA	COL1, BSP	-	NA	NA	Cell distribution ↑	

Search: ((Mesenchymal stem cells [MeSH Terms]) AND (Tissue engineering [MeSH Terms]) AND (Osteogenic OR Osteogenesis [MeSH Terms]) AND (Perfusion [MeSH Terms] OR Laminar flow) AND Bioreactors [MeSH Terms])

LTMC: Poly(L‐lactide) and Poly(L‐lactide-co-trimethylene carbonate, Col: Collagen, βTCP: Beta-tricalcium phosphate, PLA: Polylactic acid, nHA: Nano-hydroxyapatite, HA: hydroxyapatite, CaP: Calcium phosphate, PEG: Poly(ethylene glycol), PLG: Poly(lactide-co-glycolide), PLCL: Poly(l-lactide-co- ε -caprolactone), PU: Polyurethane, ECM: Extracellular matrix, HUVEC: Human umbilical vein endothelial cells, GM: Growth medium, OM: Osteogenic medium, NA: Not accessed, NC: No change

Furthermore, the high scalability of perfusion bioreactors enables the integration of other platforms, facilitating the generation of diverse stimuli, such as hydrostatic pressure and mechanical compression. For instance, the combinational application of fluid shear stress and cyclic hydrostatic pressure has been shown to promote the expression of chondrogenic markers synergistically, making it a promising approach for osteochondral regeneration [142]. Although the potential additive or synergistic effect on osteogenic differentiation remains less unexplored, pressurized environment is well acknowledged as a favorable stimulus for bone regeneration [143]. Moreover, increased hydrostatic pressure serves the additional benefit of suppressing air bubble formation during perfusion, thereby mitigating operational errors [92]. Similarly, a perfusion bioreactor integrated with a mechanical compression module has been introduced, revealing a distinct expression pattern of osteogenic markers compared to perfusion alone [111], [144].

## 6 Challenges in bridging between dynamic cell culture platform and biology

Tissue engineering has witnessed substantial progress in recent years, driven by innovative dynamic cell culture platforms that seek to mimic the complex biological microenvironments. However, it is unavoidable to shed light on the challenges faced in effectively bridging the gap between these dynamic cell culture platforms and biological systems.

In bioreactor-based bone tissue engineering, numerous challenges hinder the meaningful comparison of results across different studies. A primary obstacle lies in the vast array of variables that influence the outcomes, rendering direct comparisons nearly impossible. Factors such as cell type, donor characteristics (including age and sex) and variations, scaffold geometry, material type, and the choice of medium supplements introduce considerable variation in experimental setups. For example, the outcomes of bioreactor applications are influenced by the porous nature of scaffolds, even though an applied magnitude of flow to cells was equal. Smaller pores (e.g., < 200 µm) are associated with enhanced mass transfer when using flow-based bioreactors, while larger pores provide the main advantage of mechanical stimuli on cells due to increased flow velocity [145], [146], [147]. Additionally, scaffold-flow interaction and its association with mass transfer can be further influenced by surface microtopography and surface chemistry [145], [146], [148]. The nature of dynamic culture systems further adds complexity, as variations in flow type, flow magnitude, other accessory mechanical stimuli, and nutrient gradients influence biological events. These factors may interact each other additively, synergistically or antagonistically [92]. Unlimited combination of different parameters forces researchers to face the daunting task of navigating through this complexity and establishing a consensus on key parameters, but solving the task seems inevitable to enable a more cohesive and reliable advancement in bioreactor-based bone tissue engineering approaches.

To link between mechanical stimuli applied in dynamic cell culture platforms and biological events, the accurate description of flow characteristics is needed. In the realm of tissue engineering, computational fluid dynamics (CFD) simulations play a crucial role in understanding and predicting fluid-induced stimuli within bioreactor systems as well as designing bioreactors and scaffold geometries [149]. However, one major challenge arises from the inherent complexity and computational expense of simulating fluid behavior in three-dimensional porous scaffolds and cellular environments [150], [151]. The intricate geometries of tissue scaffolds and the presence of cells further exacerbate the computational burden, necessitating high-performance computing resources. Moreover, accurately estimating fluid stimuli, such as shear stress and fluid velocity, at the cellular level requires overcoming significant technical hurdles. The dynamic interactions between the fluid flow and cells, including fluid-structure interactions, cell deformation and spatiotemporal change in pore size and geometry due to cell growth, add another layer of complexity to the simulations. This would probably explain the reasons behind the limited provision of comprehensive descriptions of fluid stimuli applied to cells during dynamic cell culture in recent studies with complex systems. Thus, the development of efficient and accurate computational techniques for fluid simulation and estimation of fluid stimuli remains an active area of research in tissue engineering, crucial for advancing our understanding and optimizing dynamic cell culture strategies for bone tissue engineering.

## 7 Future perspectives and conclusion remarks

The accurate understanding of biophysics in bone has successfully been translated into the development of 3D cell culture systems with the aim of applying them in regenerative medicine. Most systems have adopted fluid flow to optimize mass transfer and provide fluid shear stress as it is a primary biophysical stimulus in bone and bone marrow [22]. Interstitial fluid flow is generally considered constant directional flow with uniform velocity [152]. However, in bone, interstitial fluid flow may vary in direction and magnitude in relation to physical exercises, which is altered by compressive or tensile forces on the bone matrices [8]. Therefore, to achieve a more biophysically relevant environment in vitro, dynamic culture conditions need further optimization, which includes magnitude (i.e., velocity), duration, and frequency. Moreover, development of advanced perfusion bioreactor systems that integrate other biophysical stimulus, such as mechanical compression, tension, torsion, atmospheric or hydrostatic pressure, may offer additive or synergetic effects on the regenerative capacities of engineered constructs.

The success of tissue engineering-based therapy depends on the survival of engineered constructs after transplantation and their integration to the host tissue. During in vitro cell expansion, bioreactor systems offer robust fluid flow, ensuring efficient mass transport within the construct while stimulating cell functionalities. However, after transplantation, attaining an equally robust flow within the host tissue may not be easily achievable, potentially leading to the deterioration of implanted constructs. This concern is particularly relevant for larger constructs, where passive diffusion alone proves inadequate [92]. To overcome this limitation, fluid paths need to be meticulously optimized both from material point and cellular point of views. Firstly, scaffolds need to possess a hollow guiding structure with appropriate macro- and micro-porous geometry [146], [153]. This not only facilitates the homogeneous flow passing through the scaffolds during in vitro culture but also allows for rapid blood infiltration into the constructs after transplantation, occurring prior to the establishment of a newly regenerated capillary network. In addition, co-culturing MSCs with vascular endothelial cells on/in scaffolds emerges as a promising strategy to generate microvascular network within the scaffolds. Dynamic culture under flow facilitates the capillary formation of the endothelial cells, fostering the development of a functional vascular network within the scaffolds [154], [155]. Consequently, upon implantation, the prevascularized constructs potentially improve post-transplantation vascularization and integration, increasing the survival rate of the construct and the overall success rate of the cell therapy [156]. The considerations represent a critical step towards bridging the gap between in vitro culture and successful in vivo integration, thereby advancing the field of tissue engineering and regenerative medicine.

Finally, in order to bring engineered constructs from bioreactors to patients, there are several regulatory challenges that must be overcome. MSCs used in cell therapies are classified as advanced therapy medicinal products (ATMPs), and as such, the production process for tissue engineering constructs is strictly regulated by Current Good Manufacturing Practice (cGMP). Cells manipulated in bioreactor systems are subject to strict regulatory oversight. Additionally, bioreactor systems for cell production are classified as class 1 medical devices (according to EU MDR for the EU, FDA for the USA), and must therefore satisfy regional regulations through general safety and performance tests and clinical evaluation. However, as an emerging technology, there is a lack of consensus on standards relating to device designs, safety assessment, cell production processes, and methods for evaluating produced constructs [89]. The bioreactor prototypes introduced to date have been developed under various concepts and are mostly based on tailored components, thus being potentially challenging to scale their production. Furthermore, efforts to automate cell culture are ongoing in order to decrease operator dependence and enhance production efficiency [157], [158]. Besides technical challenges, the biological inter-donor variability is also of a concern as cells do not behave equally when taken from different donors. Therefore, it is required to develop robust and standardized processes and, even more importantly, to monitor the constructs real-time during production. Ideally, the latter should encompass non-invasive monitoring of important environmental and cellular parameters (e.g., glucose concentration, oxygenation, pH, cell growth, viability, mutation, and differentiation markers). Additionally, conditions during the construct production might differ from after transplantation, making further understanding of the biological and biophysical environment in vitro and in vivo essential. In case of a centralized production (i.e., when the constructs are produced in elsewhere from where surgery is performed), strategies are needed to ensure sufficient nutrient supply during transport to the patient. This can be achieved by developing mobile, energy-independent incubator systems to maintain dynamic conditions. Consequently, the environment surrounding machinery-based tissue engineering is becoming increasingly complex. Standardization of emerging technologies can be accomplished through anticipatory consensus standards, as previously proposed and agreed upon by a nanotechnology society that included academics, clinicians, industry professionals, and regulatory bodies [159]. While the degree of standardization is subject to debate, it is likely to facilitate the commercialization and clinical translation of the technology.

## CRediT authorship contribution statement

**Shuntaro Yamada:** Conceptualization, Methodology, Resources, Investigation, Data curation, Writing – original draft, Writing – review & editing, Visualization. **Philipp Niklas Ockermann:** Investigation, Writing – original draft, Writing – review & editing. **Thomas Schwarz:** Methodology, Resources. **Kamal Mustafa:** Conceptualization, Supervision, Funding acquisition, Project administration. **Jan Hansmann:** Conceptualization, Resources, Writing – original draft, Writing – review & editing, Supervision, Project administration.

## Declaration of Competing Interest

The authors declare no potential conflict of interest with respect to the authorship and/or publication of this article.
